# Cholesterol Retards Senescence in Bone Marrow Mesenchymal Stem Cells by Modulating Autophagy and ROS/p53/p21^Cip1/Waf1^ Pathway

**DOI:** 10.1155/2016/7524308

**Published:** 2016-09-15

**Authors:** Mingyu Zhang, Yue Du, Renzhong Lu, You Shu, Wei Zhao, Zhuoyun Li, Yu Zhang, Ruixue Liu, Ti Yang, Shenjian Luo, Ming Gao, Yue Zhang, Guiye Zhang, Jiaqi Liu, Yanjie Lu

**Affiliations:** ^1^Department of Pharmacology (State-Province Key Laboratories of Biomedicine-Pharmaceutics of China, Key Laboratory of Cardiovascular Research, Ministry of Education), College of Pharmacy, Harbin Medical University, Harbin, Heilongjiang 150081, China; ^2^China Northern Translational Medicine Research and Cooperation Center, Heilongjiang Academy of Medical Sciences, Harbin Medical University, Harbin, Heilongjiang 150081, China

## Abstract

In the present study, we demonstrated that bone marrow mesenchymal stem cells (BMSCs) of the 3rd passage displayed the senescence-associated phenotypes characterized with increased activity of SA-*β*-gal, altered autophagy, and increased G1 cell cycle arrest, ROS production, and expression of p53 and p21^Cip1/Waf1^ compared with BMSCs of the 1st passage. Cholesterol (CH) reduced the number of SA-*β*-gal positive cells in a dose-dependent manner in aging BMSCs induced by H_2_O_2_ and the 3rd passage BMSCs. Moreover, CH inhibited the production of ROS and expression of p53 and p21^Cip1/Waf1^ in both cellular senescence models and decreased the percentage of BMSCs in G1 cell cycle in the 3rd passage BMSCs. CH prevented the increase in SA-*β*-gal positive cells induced by RITA (reactivation of p53 and induction of tumor cell apoptosis, a p53 activator) or 3-MA (3-methyladenine, an autophagy inhibitor). Our results indicate that CH not only is a structural component of cell membrane but also functionally contributes to regulating cellular senescence by modulating cell cycle, autophagy, and the ROS/p53/p21^Cip1/Waf1^ signaling pathway.

## 1. Introduction

Cholesterol (CH) is essential for normal cellular function not only because it is a main structural component of the cell membrane but also because it is also involved in many functional processes via regulating cellular signaling pathways, such as cell proliferation, differentiation, and death [1]. Notably, CH has been documented to promote cell proliferation [2] and protect bone marrow mesenchymal stem cells (BMSCs) from apoptosis [3].

Senescence is a complex pathophysiological process determined by a variety of factors, such as shortening of telomere length [4], oxidative stress [5, 6], and other signaling pathways involved in aging process. The cellular premature senescence is characterized by G1 cell cycle arrest, activation of cyclin-dependent kinase inhibitors including p53 and p21^Cip1/Waf1^, enhanced activity of senescence-associated *β*-galactosidase (SA-*β*-gal) [7], and reduction of autophagy [8, 9]. Reactive oxygen species (ROS) are endogenous products of metabolism and essential second messages in cellular signaling involving cell proliferation, cell cycle arrest, and cell death [10]. However, excessive ROS accumulation can damage cellular proteins, lipids, and DNA leading to cell dysfunction, senescence, and even cell death [11, 12]. Additionally, as a target of ROS, cyclin-dependent kinase inhibitor p53 is also involved in the process of cellular senescence [13]. p21^Cip1/Waf1^, one of downstream molecules of the p53 signaling pathway, plays an important role in cell proliferation and cell senescence through mediating G1-phase cell cycle arrest [14]. It has been known that with increasing cell serial passages, BMSCs display the senescence-associated phenotypes as a result of degradation of cellular organelles [15]. It is thus plausible that delaying cell senescence can enhance viability and cellular function of BMSCs.

We therefore investigated whether CH could affect senescence process of BMSCs. Our study revealed that CH elicited significant antisenescence effects in aging BMSCs at the 3rd passage or induced by H_2_O_2_ [16], likely through suppressing the ROS/p53/p21^Cip1/Waf1^ signaling pathway and regulating autophagy process. These data provide new insight into the role of CH in delaying senescence and enhancing the viability of BMSCs.

## 2. Materials and Methods

### 2.1. Animals

Healthy 2-month-old male Sprague Dawley (SD) rats were used in the present study. Rats were kept under standard animal room conditions (temperature 21 ± 1°C; humidity 55–60%) with food and water available* ad libitum* for 1 week before the experiment. All experimental procedures were in accordance with and approved by the Institutional Animal Care and Use Committee of Harbin Medical University.

### 2.2. Isolation and Culture of BMSCs

BMSCs were isolated as previously described [17]. After rats (weighing 70 ± 20 g) were anesthetized with pentobarbital (40 mg/kg, i.v.), their femurs and tibias were taken, and bone marrow cells were flushed out into culture flasks with culture medium special for BMSCs (Stem Cell Technologies Inc., Vancouver, BC, Canada) and then cultured in the BMSCs medium supplemented with 10% FBS (Stem Cell Technologies Inc., Vancouver, BC, Canada) and 5% CO_2_ at 37°C. The medium was changed every 3 days. When reaching 80% confluence, the cells were passaged at a 1 : 2 ratio. All experiments were performed using cells of the 1st to 3rd passage.

### 2.3. Cell Treatment with H_2_O_2_

The 1st passage BMSCs were plated in 96-well plates. Following attachment to the bottom, cells were incubated with varying concentrations of H_2_O_2_ (25, 50, 75, 100 *μ*M) for 6 h. Cell viability was evaluated by 3-(4, 5-dimethyl-2-thiazolyl)-2, 5-diphenyl-2-H-tetrazolium bromide (MTT). After H_2_O_2_ treatment, 20 *μ*L of MTT diluent (5 mg/mL) (Sigma-Aldrich, St. Louis, MO, USA) was added to each well and incubated with the cells for 4 h at 37°C. The medium was removed and the cells were dissolved in 150 *μ*L DMSO. The plate was shaken for 10 min for solubilization of crystals and the optical density of each well was determined at 490 nm with a microplate reader (model 680; Bio-Rad, Hercules, CA, USA).

### 2.4. In Situ *β*-Galactosidase Staining of BMSCs

Expression of senescence-associated *β*-galactosidase (SA-*β*-gal) in BMSCs was analyzed using *β*-galactosidase reporter gene staining kit (Genmed Scientifics Inc., Plymouth, MN, USA), according to the manufacturer's instructions. After staining, the cells were examined under a microscope and SA-*β*-gal positive cells were counted and calculated.

### 2.5. Cell Cycle Analysis

Cell cycle status was analyzed using propidium iodide (PI, Sigma-Aldrich, St. Louis, MO, USA) staining. Briefly, cells were cultured in serum-free medium for 24 h for growth synchronization. Then they were treated with different concentrations of CH for 30 h. After two washes with ice-cold PBS, the adhered cells were collected and fixed in 75% ethanol overnight at 4°C and incubated with a mixture of 50 *μ*g/mL PI and 25 *μ*g/mL RNase A (Sigma-Aldrich, St. Louis, MO, USA) in the dark at 37°C for 30 min. Finally, the samples were analyzed by flow cytometry (BD Biosciences, San Jose, CA, USA) and the proportion of cells in G1, S, and G2 phases was measured.

### 2.6. Detection of ROS Level

Level of intracellular ROS was measured with the reactive oxygen species assay kit (Beyotime Institute of Biotechnology, Shanghai, China). After wash and centrifugation, BMSCs were treated with fluorescent probes DCFH-DA (1 : 1000) and incubated at 37°C for 20 min. The fluorescence intensity was measured using a microplate reader (Bio-Rad, Hercules, CA, USA) under 488 nm excitation wavelength and 525 nm emission wavelength.

### 2.7. Western Blotting

Total protein was extracted from BMSCs for immunoblotting analysis. Briefly, protein content was determined with a bicinchoninic acid protein assay kit (Beyotime Institute of Biotechnology, Shanghai, China) using bovine serum albumin as the standard. Protein samples (60–80 *μ*g) were separated in 15% SDS-PAGE and blotted to nitrocellulose membranes. After blocked with 5% nonfat milk, the membranes were probed with p21^Cip1/Waf1^ (BD Pharmingen*™*, Franklin lakes, NJ, USA), p53 (Cell Signaling Technology, Danvers, MA, USA), LC3 (Sigma-Aldrich, Saint Louis, MO, USA), or GAPDH antibodies (Kangcheng Inc., Shanghai, China) in 5% nonfat milk and incubated overnight at 4°C. Infrared fluorescent dye-labeled secondary antibody (Alexa Fluor, Molecular Probes, Eugene, OR, USA) was incubated with the membrane for 1 h. Western blot bands were collected using Infrared Imaging System (LI-COR Biosciences, Lincoln, NE, USA) and the band density was quantified using Odyssey 3.0 software for each group and normalized by GAPDH.

### 2.8. Statistical Analysis

Data are presented as mean ± SD. Statistical comparisons among multiple groups were performed by analysis of variance (ANOVA) followed by Tukey's multiple comparison test. Statistical values were calculated using the SPSS 19.0 software and illustrated using the GraphPad Prism 5.0. Differences with a value of *P* < 0.05 were regarded as statistically significant.

## 3. Results

### 3.1. Development of Senescence-Associated Phenotypes in BMSCs following Serial Passages

**Figure 1:**
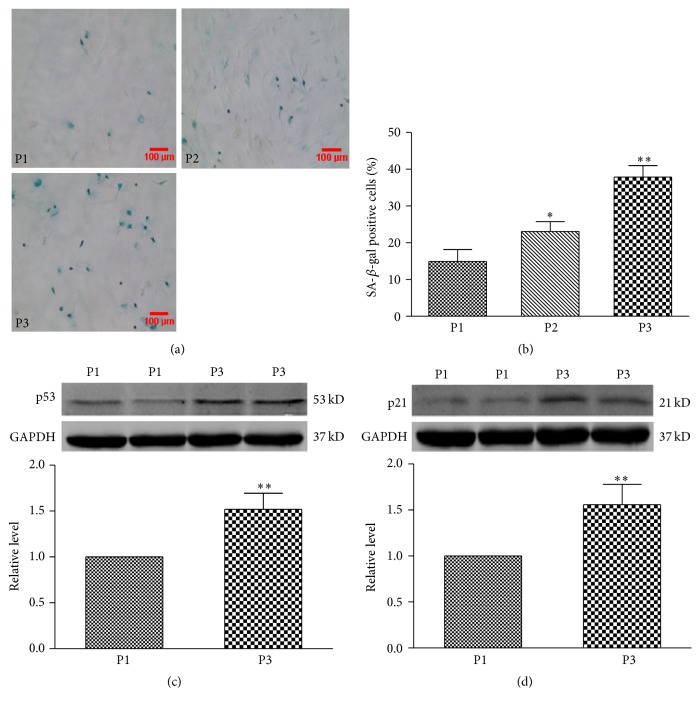
The detection of senescence-associated phenotype in BMSCs at the 1st (P1) and 2nd (P2) and 3rd passage (P3). (a) Representative image of *β*-galactosidase (SA-*β*-gal) staining in BMSCs at P1, P2, and P3. (b) Percentage of SA-*β*-gal positive senescent cells in BMSCs at P1, P2, and P3. (c) and (d) Expression levels of p53 and p21^Cip1/Waf1^ proteins in BMSCs at P1 and P3. Data are expressed as mean ± SD, *n* = 3 for each group, ^*∗*^ *P* < 0.05, ^*∗∗*^ *P* < 0.01 versus P1.

Cultured BMSCs displayed the senescent phenotypes in a passage-dependent manner, characterized by increased number of senescence-associated *β*-galactosidase (SA-*β*-gal) positive cells from 14.9 ± 3.3% in the 1st passage BMSCs to 23.1 ± 2.7% and 37.8 ± 3.1% in the 2nd and 3rd passages, respectively (*P* < 0.01; Figures 1(a) and 1(b)). SA-*β*-gal is a widely used biomarker for ageing and senescent mammalian cells [16]. In addition, we examined the expression of senescence-related proteins p53 and p21^Cip1/Waf1^. The results showed that p53 and p21^Cip1/Waf1^ were both markedly increased in their expression in the 3rd passage BMSCs compared with the 1st passage BMSCs (*P* < 0.01; Figures 1(c) and 1(d)). These results indicate that BMSCs can develop the senescent phenotypes in a passage-dependent manner.

### 3.2. CH Reduces Senescence-Associated SA-*β*-gal Activity and ROS Level in Senescent BMSCs

**Figure 2:**
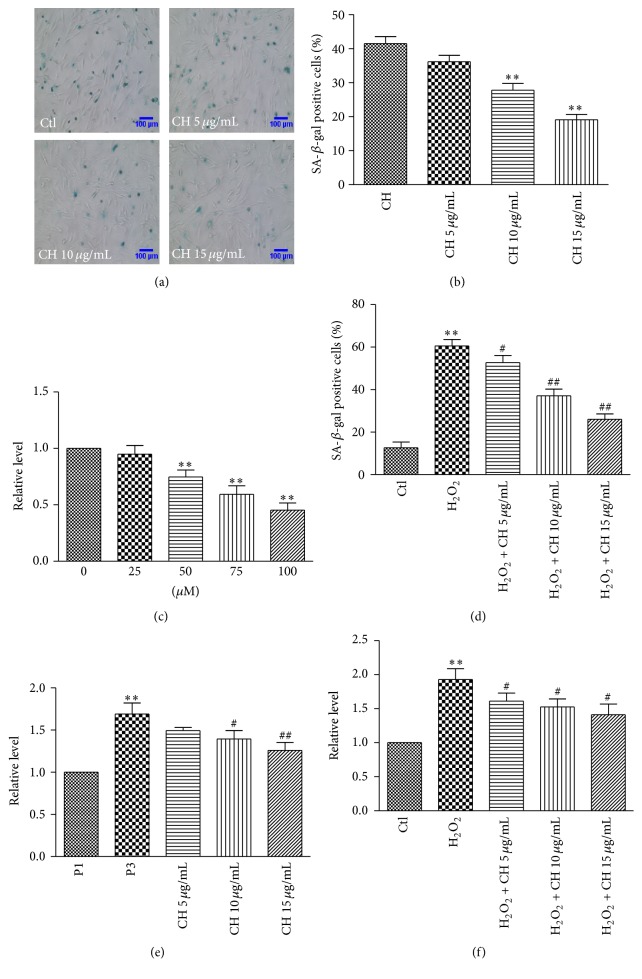
The detection of SA-*β*-gal activity and ROS level in BMSCs. (a) Representative image of SA-*β*-gal staining in the P3 BMSCs in the absence (control, Ctl) or presence of CH (5, 10, and 15 *μ*g/mL). (b) Percentage of SA-*β*-gal positive senescent cells in different groups shown in (a). *n* = 3 for each group, ^*∗∗*^ *P* < 0.01 versus Ctl group. (c) MTT results of BMSCs with a variety of concentration of H_2_O_2_. *n* = 8 for each group, ^*∗∗*^ *P* < 0.01 versus Ctl group. (d) Percentage of SA-*β*-gal positive cells in the P1 BMSCs treated with H_2_O_2_ or H_2_O_2_ + different concentrations of CH. *n* = 3 for each group, ^*∗∗*^ *P* < 0.01 versus Ctl group, ^#^ *P* < 0.05, ^##^ *P* < 0.01 versus H_2_O_2_ group. (e) ROS level in BMSCs in different groups. *n* = 3 for each group, ^*∗∗*^ *P* < 0.01 versus P1 group, ^#^ *P* < 0.05, ^##^ *P* < 0.01 versus P3 group. (f) ROS level in the BMSCs treated with H_2_O_2_ or H_2_O_2_ + different concentrations of CH. *n* = 3 for each group, ^*∗∗*^ *P* < 0.01 versus Ctl group, ^#^ *P* < 0.05 versus H_2_O_2_ group.

The 3rd passage BMSCs were incubated in the absence or presence of cholesterol (CH) at 5, 10, or 15 *μ*g/mL for 48 h. The number of SA-*β*-gal positive cells was significantly decreased in the BMSCs treated with 10 and 15 *μ*g/mL CH compared with the control group (no treatment) (*P* < 0.01; Figures 2(a) and 2(b)). CH at 5 *μ*g/mL did not significantly affect the number of SA-*β*-gal positive cells.

To confirm the above results, we used H_2_O_2_ to induce senescence [18] as another model to further evaluate the antisenescence effect of CH. We incubated BMSCs of the 1st passage with varying concentrations of H_2_O_2_ (25, 50, and 100 *μ*M) for 6 h to induce oxidative stress and senescence. Our MTT assay showed that 50 and 100 *μ*M H_2_O_2_ damaged the viability of BMSCs, but 25 *μ*M H_2_O_2_ failed to affect cell survival (Figure 2(c)). Hence, 25 *μ*M H_2_O_2_ was selected to induce senescence of the 1st passage BMSCs in our subsequent experiments. BMSCs were treated with H_2_O_2_ alone or combined with CH at 5, 10, or 15 *μ*g/mL. After 6 h of 25 *μ*M H_2_O_2_, BMSCs were cultured with CH for 48 h. The SA-*β*-gal positive cells in H_2_O_2_ group were significantly increased compared with the control group (*P* < 0.01). However, CH reduced the positive cells induced by H_2_O_2_ (Figure 2(d)).

To investigate the effect of CH on production of ROS in senescent BMSCs, the 3rd passage BMSCs were cultured alone or were incubated with CH at 5, 10, or 15 *μ*g/mL for 48 h. The results showed that the ROS level was significantly enhanced in the 3rd passage BMSCs compared with the 1st passage group, and this increase was attenuated by 10 and 15 *μ*g/mL CH (Figure 2(e)). We also incubated cells with 25 *μ*M H_2_O_2_ to enhance oxidative stress in cells. As expected, H_2_O_2_ increased ROS level in BMSCs compared with the cells without H_2_O_2_ treatment. Notably, CH decreased the ROS level at all tested concentrations (Figure 2(f)).

### 3.3. CH Decreases the Expression of Senescence-Related Proteins p53 and p21^Cip1/Waf1^ and Autophagy-Related Protein LC3

**Figure 3:**
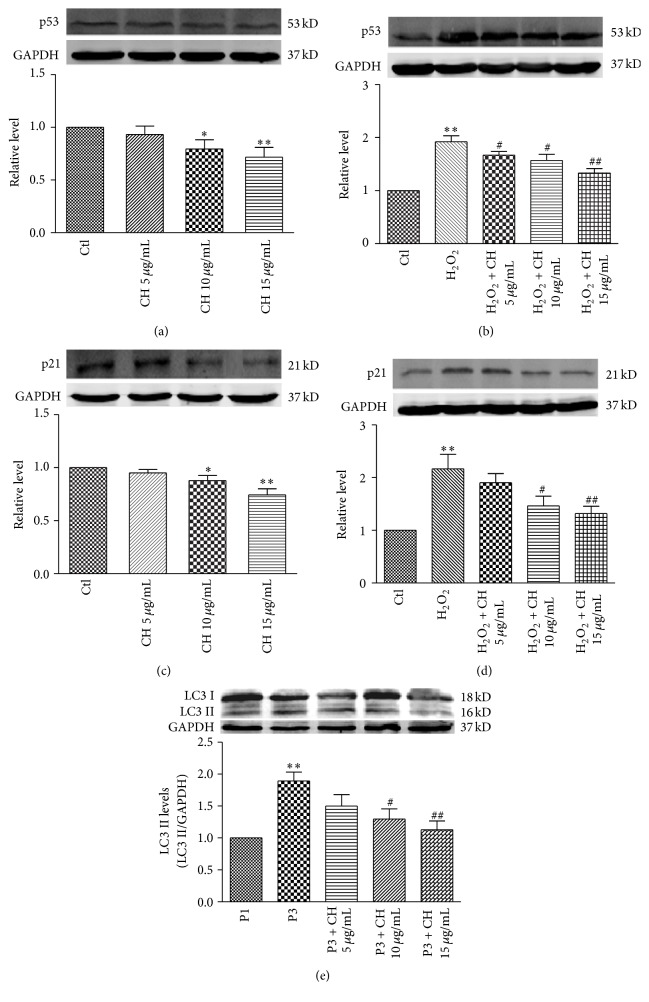
Effects of CH on expression of p53, p21^Cip1/Waf1^, and LC3 proteins. (a) and (b), Expression of p53 protein in the P3 or in the P1 with H_2_O_2_ (25 *μ*M, 6 h). *n* = 3 for each group, ^*∗*^ *P* < 0.05, ^*∗∗*^ *P* < 0.01 versus Ctl group; ^#^ *P* < 0.05, ^##^ *P* < 0.01 versus H_2_O_2_ group. (c) and (d) Expression of p21^Cip1/Waf1^ proteins in the P3 or in the P1 cells treated with H_2_O_2_. *n* = 3 for each group, ^*∗*^ *P* < 0.05, ^*∗∗*^ *P* < 0.01 versus Ctl group; ^#^ *P* < 0.05, ^##^ *P* < 0.01 versus H_2_O_2_ group. (e) Expression of LC3 protein in BMSCs with or without different concentrations of CH. *n* = 3 for each group, ^*∗∗*^ *P* < 0.01 versus P1 group; ^#^ *P* < 0.05, ^##^ *P* < 0.01 versus P3.

We further examined the effects of CH on expression of senescence-related proteins p53 and p21^Cip1/Waf1^ and autophagy-related protein LC3 (Microtubule-Associated Protein 1 Light Chain 3, an autophagy marker). Western blot analysis showed that p53, p21^Cip1/Waf1^, and LC3 were all significantly decreased at the protein level in the 3rd passage BMSCs incubated with CH compared with the control group (Figures 3(a), 3(c), and 3(e)). We also investigated the effects of CH on expression of p53 and p21^Cip1/Waf1^ in senescent BMSCs induced by oxidative stress. Western blot results showed that p53 and p21^Cip1/Waf1^ protein levels were significantly increased by H_2_O_2_ (25 *μ*M for 6 h) compared with control, and these increases were inhibited by CH in a dose-dependent manner (Figures 3(b) and 3(d)).

### 3.4. CH Attenuates BMSCs Senescence through the p53 Pathway and Autophagy Process

**Figure 4:**
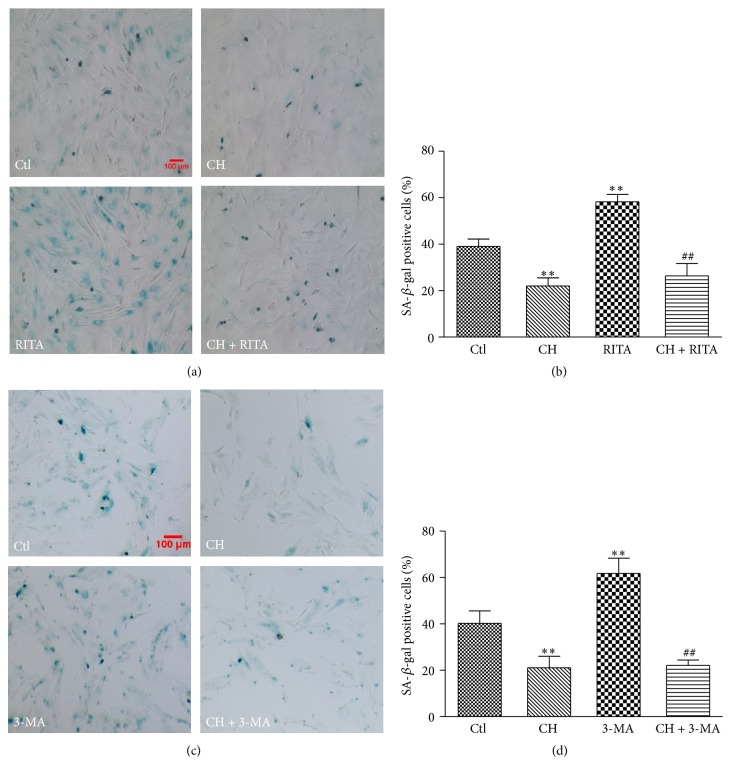
Activity of SA-*β*-gal in BMSCs with RITA (p53 activator) or 3-MA (autophagy inhibitor). (a) Representative image of SA-*β*-gal staining in the P3 BMSCs treated with RITA a p53 activator (1.0 *μ*M), CH (15 *μ*g/mL), or RITA + CH. (b) Percentage of SA-*β*-gal positive cells in different groups shown in (a). *n* = 3 for each group, ^*∗∗*^ *P* < 0.01 versus Ctl group; ^##^ *P* < 0.01 versus RITA group. (c) Representative image of SA-*β*-gal staining in the P3 BMSCs treated with 3-MA an autophagy inhibitor (5.0 mM), CH (15 *μ*g/mL), or 3-MA + CH. (d) Percentage of SA-*β*-gal positive cells in different groups shown in (c). *n* = 3 for each group, ^*∗∗*^ *P* < 0.01 versus Ctl group; ^##^ *P* < 0.01 versus 3-MA group.

To investigate whether the p53 pathway was involved in the antisenescence effects of CH in the aging BMSCs, a p53 activator RITA (reactivation of p53 and induction of tumor cell apoptosis; Santa Cruz Biotechnology, Inc., Dallas, TX, USA) was used to evaluate the role of p53 in this process. The number of the SA-*β*-gal positive cells was significantly increased in the BMSCs treated with RITA (1.0 *μ*M for 12 h). However, RITA failed to increase the rate of SA-*β*-gal positive cells in the 3rd passage BMSCs after CH (15 *μ*g/mL) pretreatment for 9 h (Figures 4(a) and 4(b)). The results suggest that the p53 pathway is involved in antisenescence effects of CH in BMSCs.

In addition, we examined whether autophagy process contributed to the regulation of senescence by CH. 3-MA (3-methyladenine), an autophagy inhibitor (Sigma-Aldrich, Saint Louis, MO, USA) was used in the experiment. The SA-*β*-gal staining results showed that the positive cells were significantly increased in the BMSCs treated with 3-MA (5.0 mM for 24 h). After CH (15 *μ*g/mL) pretreatment for 9 h, 3-MA failed to increase the number of SA-*β*-gal positive cells (Figures 4(c) and 4(d)). Similarly, the results indicated that autophagy was also involved in the antisenescence effect of CH in BMSCs.

### 3.5. CH Inhibits the G1 Cell Cycle Arrest of Senescent BMSCs

**Figure 5:**
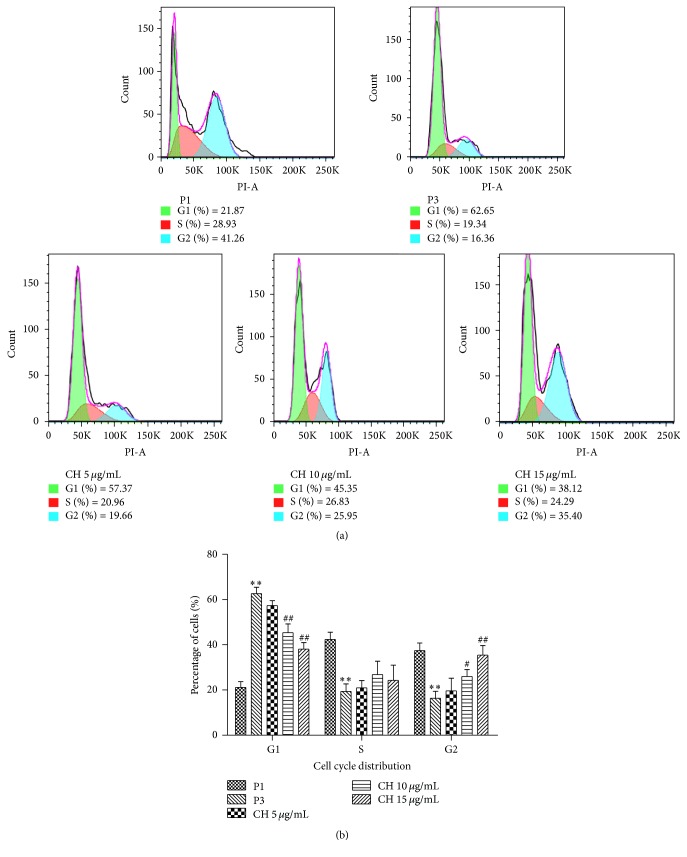
Influences of CH on cell cycle of BMSCs. (a) Analysis of cell cycle distribution by flow cytometry in P1 and P3 in the absence or presence of CH 5, 10, and 15 *μ*g/mL. (b) Statistical results of percentage of cells in different cell cycle phases. *n* = 3 for each group, ^*∗∗*^ *P* < 0.01 versus P1; ^#^ *P* < 0.05, ^##^ *P* < 0.01 versus P3.

Senescence is often marked by a decrease in cell proliferation and an increase in G1 cell cycle arrest. Thus we analyzed the percentage of cells in G1 cell cycle of the 3rd passage BMSCs with varying concentrations of CH for 48 h. As shown in Figures 5(a) and 5(b), cells in G1 phase were increased in the 3rd passage in the absence of exogenous CH, and with CH treatment, concentration-dependent decreases of cells in G1 phase and corresponding increases in the percentage of cell in G2 phase were consistently observed.

## 4. Discussion

In the present study, we demonstrated that CH possessed significant antisenescence property in aging BMSCs at the 3rd passage or under oxidative stress and the beneficial actions were likely conferred by the ability of CH to suppress the ROS/p53/p21^Cip1/Waf1^ pathway and to regulate the autophagy process. Three lines of evidence were generated. First, CH reduced the number of SA-*β*-gal positive BMSCs at the 3rd passage or in the presence of H_2_O_2_ in a dose-dependent manner. Second, CH restored the increased levels of ROS, p53, and p21^Cip1/Waf1^ in aging BMSCs and effects of CH on the number of SA-*β*-gal positive BMSCs were mediated through the regulation of autophagy and the ROS/p53/p21^Cip1/Waf1^ pathway, as demonstrated by the administration of RITA (a p53 activator), and 3-MA (autophagy modulator). Third, CH attenuated G1 cell cycle arrest and increased percentage of the cells in G2 phase in aging BMSCs. Our findings therefore unraveled a novel cellular function of CH and the underlying mechanism.

BMSCs possess multiple biological functions, but their survival and cellular functions are declined along with cell senescence. Studies have reported that BMSCs demonstrated senescence changes and declining of cell function with cell serial passages [15]. In the present study we found that BMSCs at the 3rd passage of culture displayed senescence-associated phenotype characterized by increased SA-*β*-gal positive cells, expression of senescence-associated proteins p53 and p21^Cip1/Waf1^, increase of cells in G1 phase, and altered autophagy. As a potent inducer of the p53/p21^Cip1/Waf1^ pathway, ROS was also increased in the 3rd passage BMSCs. Notably, CH significantly decreased SA-*β*-gal positive cells in a dose-dependent manner, regulated the autophagy process, and attenuated the level of ROS and expression of p53 and p21^Cip1/Waf1^ in aging BMSCs.

Autophagy is a conserved mechanism from yeast to humans for the maintenance of cellular homeostasis through cytoplasmic and organelle turnover [19]. A number of studies reported that there is an intimate connection between autophagy and senescence, and autophagy plays an important role in repairing cell damage and delaying cell senescence[8, 20, 21]. Our data showed that the expression of the autophagy marker protein LC3 was significantly increased in senescent BMSCs. While this result might suggest enhancement of autophagy as a causal factor or a contributor to BMSC senescence, other evidence argues against this view. First, the increase in LC3 level was mitigated by CH, along with a reversal of BMSC senescence. Second, 3-MA, an autophagy inhibitor, increased the number of SA-*β*-gal positive cells, indicating suppression of autophagy is linked to senescence development in BMSCs. Moreover, the 3-MA-mediated senescence of BMSCs was considerably retarded by CH, further suggesting that restoring autophagy protects against senescence process. A report showed that 3-MA can act either as an autophagy inhibitor or as an autophagy activator depending on specific cellular context/conditions [22]. In our study, 3-MA likely acted as an autophagy inhibitor to aggravate cell senescence, but not an autophagy activator. Consistently, CH treatment restored the suppressed autophagy and relieved the 3-MA-induced cell senescence, suggesting that the mechanism for the antisenescence property of CH is related to the regulation of autophagy in BMSCs. We therefore attempted to speculate that enhanced autophagy is a compensatory mechanism to counteracting the senescence of BMSCs, and CH downregulated LC3 expression likely through inhibiting senescence and attenuating the compensatory action of autophagy.

CH, the major component of cell membranes, is essential for normal cellular functions and also plays a role in a variety of biological processes. Distribution and function of CH are different among cells and tissues [23]. However, high level of CH can cause cell or tissue damage; for example, 60 *μ*g/mL CH evokes damaging effects in pancreatic *β*-cell line [24] and CH 30 *μ*g/mL induces smooth muscle cells death [25]. Here we tested lower concentrations of CH (5, 10, and 15 *μ*g/mL) for its effects on the aging process in BMSCs. Our results demonstrated that CH showed the antisenescence effect on BMSCs via suppressing the ROS/p53/p21^Cip1/Waf1^ signaling pathway and regulating the autophagy process. Moderate level of CH promoted cell proliferation which might account for its antisenescent action in BMSCs. It might be that supplementation of adequate CH is essential for maintenance of normal function of cell.

In the present study, we have proved that moderate concentration of CH is beneficial for delaying senescence of BMSCs. Furthermore, we revealed the underlying mechanism of antisenescence effects of CH in BMSCs through at least the ROS/p53/p21^Cip1/Waf1^ signaling pathway and autophagy process. The results will help us better understand and broaden the biological actions of CH. However, our study investigated the effect of CH on senescent BMSCs only in vitro. Further in vivo study is required to confirm these findings.

In summary, our study suggests that CH is not only a structural component of the cell membrane, but also a functional regulator of cellular senescence by modulating cell cycle, autophagy, and the ROS/p53/p21^Cip1/Waf1^ pathway. Our data also indicate maintenance of CH at certain level is necessary for the basic function of cells.
